# Impaired quality of life in patients with systemic sclerosis compared to the general population and chronic dermatoses

**DOI:** 10.1186/1756-0500-7-594

**Published:** 2014-09-02

**Authors:** Agnes Bretterklieber, Clemens Painsi, Alexander Avian, Nora Wutte, Elisabeth Aberer

**Affiliations:** Department of Dermatology and Venereology, Medical University of Graz, Auenbrugger Platz 8, A-8036 Graz, Austria; Department of Dermatology, General Hospital, Klagenfurt, Austria; Institute for Medical Informatics, Statistics and Documentation, Medical University of Graz, Graz, Austria

**Keywords:** Systemic sclerosis, Quality of life, Lupus erythematosus, Chronic skin disease, SF-36

## Abstract

**Background:**

Systemic sclerosis (SSc) is a rare and potentially life threatening autoimmune disorder. The burden of disease compared to other dermatoses is unknown. The purpose of this study was to assess both the quality of life in patients with SSc and the variables that are associated with poor quality of life. Forty-one patients with systemic sclerosis (29 limited, 2 diffuse, 10 undifferentiated forms) were assessed with respect to their health status and compared to published data for the normal population, SSc patients from other studies, and patients with chronic skin diseases.

**Results:**

For the most part, our SSc patients had better outcomes in all 8 dimensions of the SF-36 than SSc patients from other studies, and poorer scores than the healthy population and those with occupational contact dermatitis, ichthyosis, non-melanoma skin cancer, contact dermatitis, atopic eczema, chronic nail disease, vitiligo, health care workers with work-related disease, and those with other chronic skin diseases, but significantly better scores for mental health than those with nail disease, vitiligo, and health-care workers. Patients with atopic dermatitis, psoriasis and pemphigus had significantly poorer mean scores in social function and mental health than SSc patients. Patients with pemphigus were also significantly impaired in their physical and emotional roles. Patients with systemic lupus erythematosus (SLE) had the significantly poorest mean scores for QoL in all 8 domains except bodily pain and emotional role.

**Conclusion:**

Besides SLE, SSc is one of the most severe chronic dermatologic diseases in terms of reduced QoL. Since SSc cannot be cured, treatment strategies should include therapeutic interventions such as psychotherapy, social support, physiotherapy, and spiritual care. Their beneficial effects could be studied in future.

## Background

Systemic sclerosis (SSc) is a rare and potentially life threatening autoimmune disorder accompanied by fibrosis of the skin and/or internal organs, vascular lesions, and specific antibodies [1]. The clinical appearance of the disease is polymorphous. SSc generally starts with Raynaud’s phenomenon (RP) and proceeds to limited or diffuse sclerosis of the skin. The undifferentiated form (uSSc) is diagnosed when a patient does not fulfil the American College of Rheumatology (ACR) criteria [2]. Disease progression may vary considerably and is unforeseeable. The impact of this complex disease on quality of life (QoL) and its variables has received substantial attention in recent years [3], and quality of life questionnaires to assess it have become important instruments to validate outcomes in patients treated with new modalities, such as biologics [4].

One of the most widely used instruments to assess QoL is the Medical Outcomes Study 36-Item Short Form Health Survey (SF-36). It is a general health status questionnaire, measuring patient-centered rather than biological or disease-centered outcomes. It also measures physical and mental health constructs [5, 6]. SF-36 has been used to assess QoL in rheumatic disease, and has been validated in patients with rheumatoid arthritis, psoriatic arthritis, and systemic lupus erythematosus [7].

Several studies have investigated QoL in SSc in- and outpatients in various age groups with diverse organ involvement [3, 7–13]. Twelve datasets, comprising 1127 SSc patients, were included in a systematic review to investigate health-related QoL. The latter was reduced in patients with SSc: their pooled SF-36 Physical Component Summary (PCS) scores were more than 1 standard deviation (SD) below the general population [9].

Similar to SSc, dermatoses such as psoriasis, atopic dermatitis, pemphigus, or lupus erythematosus also follow a chronic incurable course with impaired QoL. Our research question was whether patients with SSc might have a higher disease burden than those with other dermatoses. To our knowledge, such a comparison has not yet been published. Here, we compared SSc data from the SF-36 questionnaire with published data from healthy persons, SSc patients, and patients with chronic dermatoses, and analyzed clinical factors that contribute to the impairment of QoL.

## Results

### QoL in different types of SSc compared to healthy persons and to other studies on QoL in SSc

**Figure 1:**
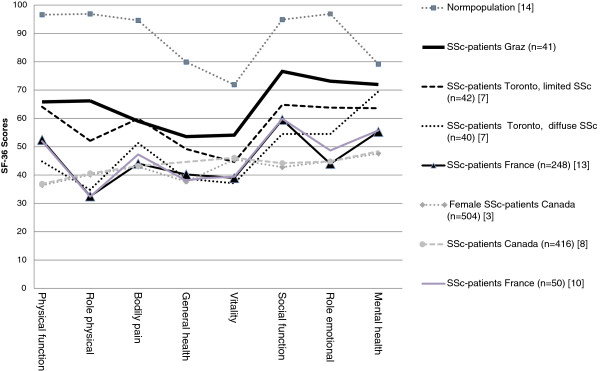
**Comparison of SF-36 values in SSc patients and healthy controls.**

QoL ranged from significantly poorer to comparable in all 8 dimensions of the SF-36 as compared to the normal population (Table 1) [14]. For the most part, our SSc patients had better outcomes in all 8 dimensions of the SF-36 than SSc patients from other studies (Figure 1).

**Table 1:** **Comparison of SF-36 items in patients with SSc and those with other dermatoses**

	Number of patients	Mean age (years)	Median disease duration (years)	Physical function	Role physical	Bodily pain	General health	Vitality	Social function	Role emotional	Mental health
Our SSc patients	41	58 ± 13	*8 (2 – 40)*	66 ± 27	66 ± 43	59 ± 26	54 ± 24	54 ± 23	77 ± 25	73 ± 42	72 ± 17
**Better or similar in all dimensions**											
Normal population without chronic disease [14]				*97*	97	*95*	*80*	*72*	*95*	97	80
Patients with occupational contact dermatitis [15]	109	41	*2.7*	*95*	*100*	*82*	*72*	*65*	*88*	*100*	*80*
Patients with ichthyosis [16]	121	44.8		*95*	*100*	*84*	*77*	60	*100*	*100*	*80*
Patients with non-melanoma cervicofacial skin cancer [17]	121	63^a^		*85*	78	*81*	*74*	*65*	86	84	76
Patients with contact eczema [18]	38			*95*	*100*	63	*72*	58	*88*	*100*	70
Patients with atopic eczema [19]	70			*88*	76	*74*	*65*	61	83	78	*75*
**Poorer only in mental health**											
Patients with nail disorders ( >1a) [20]	72			*88*	76	*77*	*65*	*64*	77	67	**65**
Patients with vitiligo in China [21]	101	30.8	*6.8*	*89*	68	*80*	*65*	*68*	76	65	**61**
Health care workers with work-related skin diseases [22]	278	36.9	*6.2*	*90*	77	65	*63*	54	81	80	**66**
Chronic skin diseases [14]				*80*	71	*69*	57	54	80	74	**65**
**Poorer in social function and mental health**											
Patients with atopic dermatitis [23]	66	34.2	*20.2*	*81*	56	*74*	*62*	54	**67**	57	**51**
Patients with different types of psoriasis < 10 years [24]	380	44.3	*12.4*	*81*	62	65	*64*	59	**62**	56	**58**
Psoriasis ≥ 10 years [24]				77	62	60	58	56	**59**	54	**56**
**Poorer or similar in all dimensions**											
Patients with pemphigus vulgaris [25]	58			72	**43**	63	49	49	**61**	**47**	**53**
Patients with systemic lupus erythematosus [26]	90	40.5	*10.4*	**50**	**37**	54	**41**	**41**	**61**	56	**62**

^a^Median.

Bold: SSc patients have significantly better outcomes, p < .05.

Italics: SSc patients have significantly poorer outcomes, p < .05.

Patient demographics such as age, sex, duration of disease and RP are shown in Table 2. We grouped diffuse and limited SSc together and compared them to uSSc because of the small sample sizes, which was one of the limitations of our study. Pitting scars were recorded in 11/31 (35%) of SSc and 1/10 (10%) of uSSc patients, digital ulcers in 6/31 (19%) of SSc patients. Six (60%) of the uSSc patients had minimal skin sclerosis distal to the metacarpo-phalangeal joints; one had pitting scars, one had lung involvement but no bihilar lung fibrosis, one had PAH, and there was one patient with gastrointestinal involvement.

**Table 2:** **Demographics and SF-36 scores in SSc patients according to the ACR-criteria classification**

	All SSc patients	Limited or diffuse SSc	Undifferentiated SSc
**Number of patients**	41	31	10
**Female**	88% (36/41)	90 % (28/31)	80% (8/10)
**Mean age (min-max)**	58 (23-84)	59.6 (33-84)	54 (23-79)
**Mean duration of SSc, years (min-max)**	9 (2-40)	10.4 (2-40)	5 (2-13)
**Mean duration of Raynaud, years (min-max)**	12 (2-40)	12.4 (2-40)	7.5 (2-25)
**Lung symptoms**	41% (17/41)	48% (15/31)	20% (2/10)
**Skin involvement**	85% (35/41)	94% (29/31)	60% (6/10)
**Raynaud phenomenon**	100% (41/41)	100% (31/31)	100% (10/10)
**Pitting scars**	29% (12/41)	35% (11/31)	10% (1/10)
**Digital ulcers**	15% (6/41)	19% (6/31)	0%
**GI-tract involvement**	37% (15/41)	45% (14/31)	10% (1/10)
**Arthralgia/arthritis**	15% (6/41)	19% (6/31)	0%
**PAH**	12% (5/41)	13% (4/31)	10% (1/10)
**Renal crisis**	2% (1/41)	3% (1/31)	0%
**Calcinosis cutis**	22% (9/41)	29% (9/31)	0%
**Karnofski index (min-max)**	77% (50-100%)	70% (50-100%)	84% (70-100%)
**Rodnan score (min-max)**	6.7 (1-31)	8.0 (1-31)	2.8 (1-6)
**SF-36 scores mean (min-max)**			
**Physical function**	65.8 (5.6-100.0)	61.1 (5.6-100.0)	71.0 (33.3-100.0)
**Physical role**	66.2 (0.0-100.0)	70.2 (0.0-100.0)	54.6 (0.0-100.0)
**Bodily pain**	59.0 (22.0-100.0)	58.7 (22.0-100.0)	60.1 (25.0-100.0)
**General health**	53.6 (15.0-97.0)	51.5 (15.0-92.0)	59.4 (18.8-100.0)
**Vitality**	54.1 (5.0-95.0)	53.3 (5.0-95.0)	56.5 (35.0-75.0)
**Social function**	76.6 (0.0-100.0)	75.9 (0.0-100.0)	78.8 (50.0-100.0)
**Emotional role**	73.1 (0.0-100.0)	76.5 (0.0-100.0)	63.0 (0.0-100.0)
**Mental health**	72.0 (28.0-100.0)	70.7 (28.0-100.0)	75.8 (52.0-88.0)

PAH: pulmonary arterial hypertension.

GI: gastrointestinal.

Clinical factors such as digital ulcers (p = 0.307), pitting scars (p = 0.231), or calcinosis (p = 0.083) were comparable for patients with uSSc and those with manifest SSc. However, physical function was significantly poorer in patients with dysphagia (p = 0.021). Patients with SSc had a higher Rodnan score (SSc mean: 8.0 range: 1 – 31 vs. uSSc mean 2.8, range: 1 – 6; p = 0.015) but comparable duration of disease (p = 0.252). The scales for bodily pain were not different for our uSSc and SSc patients (p = 0.756).

**Figure 2:**
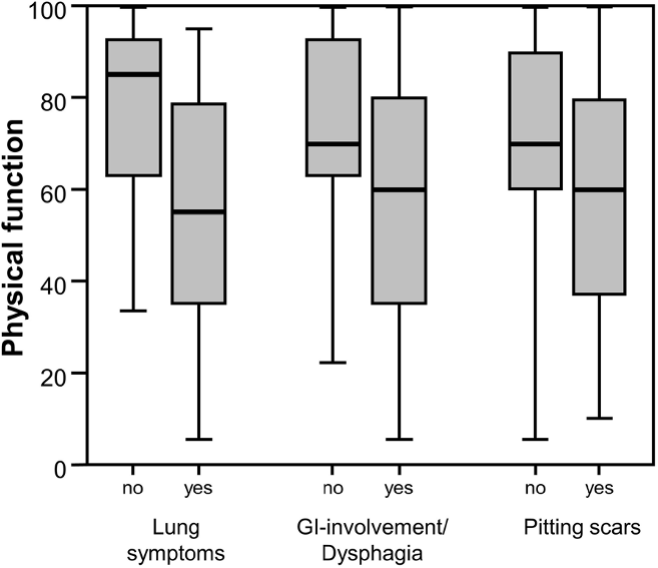
**Physical function in patients with different clinical complaints.** Significantly poorer scores were seen in patients with lung symptoms (p = 0.006), poorer scores in gastrointestinal tract involvement or dysphagia (p =0.051), but no significant differences in patients with pitting scars (p = 0.342).

Patients with or without clinical complaints independent of the SSc type were compared for pulmonary involvement, gastrointestinal tract involvement/dysphagia, and pitting scars. Physical function was significantly poorer in patients with pulmonary involvement (p = 0.006) but not in those with gastrointestinal tract involvement/dysphagia, (p =0.051) or pitting scars (p = 0.342) (Figure 2). The sample size was too small for statistical comparisons of other clinical complaints.

### Relevant findings in SSc compared to other dermatoses

Comparing SSc to other dermatoses, subscale scores for SSc in all dimensions were significantly poorer than or comparable to patients with occupational contact dermatitis [15], ichthyosis [16], non-melanoma skin cancer [17], and contact eczema [18] (Table 1). SSc patients had better results in mental health compared to patients with long-standing nail disease [20], vitiligo [21], chronic skin diseases [14], and health care workers with work related disease [22], The SF-36 scores in patients with atopic dermatitis vary and can be better or worse than in SSc [19, 23].

In comparison to SSc patients, those with psoriasis and pemphigus were significantly impaired in mental health and social function; with pemphigus this was also true for physical and emotional roles compared to SSc patients [24, 25].

Patients with SLE had lower mean scores than SSc patients for QoL in all 8 domains except bodily pain and emotional role, and poorer QoL in all 8 domains than age-matched samples from the normal female population in the US [26].

## Discussion

In this study, SSc patients were impaired in their QoL compared to healthy persons, but had better SF-36 outcome compared to SSc patients from other studies. But as in other studies, the domains of the SF-36 were too insensitive to find a significant difference in QoL between patients with uSSc and with manifest SSc [7, 10].

A very recent study from Naples determined QoL with the SF-36 in early SSc, a group comparable with our uSSc. Compared to this study, physical function and physical role were poorer in our patient cohort [12]. This may be due to the colder climate in Austria, with more severe RP during the winter and summer rainy periods. Even primary RP can lead to a significant reduction in QoL compared to controls with respect to both the physical and the mental component scores [27].

The group most comparable to our study comprised patients with limited SSc in the study by Johnson et al., in which vitality (51.1 vs. 44.6, p = 0.014), social function (76.6 vs. 64.8, p = 0.005), and mental health (80.0 vs. 63.6, p = 0.005) were significantly poorer than in our SSc patients, whereas physical function and bodily pain scores were not significantly different (Figure 1) [7]. They also studied patients with diffuse SSc, who were similar to our patients only in mental health but worse in the other dimensions. Also in the study by Perrot et al., pain dimension scores are significantly lower than in ours [28]. A neuropathic component is associated with a higher pain score.

In a recent cross-sectional survey of 381 SSc patients that was focused on anxiety and depression, 40.3% of them had diffuse cutaneous SSc, 50.5% limited cutaneous SSc and 9.2% limited SSc (uSSC in our definition) [29]. Poorer mental health was significantly associated with anxiety and depression. This was based on a poorer physical component score and related to disabilities, especially of the hands, and aesthetic impairment and not to organ involvement [29]. We did not investigate depression and anxiety. Our patients had impaired median physical function compared to the normal population (65.8 vs. 97), but better physical function compared to Nguyen’s study involving the hands on the basis of digital ulcers (15 vs. 45.3%), pitting scars (29 vs. 58.8%) and calcinosis (22 vs. 33.7%).

All the other SSc studies using the SF-36 scales revealed significantly poorer scores in QoL than in our study (Figure 1) [3, 8, 10, 13]. Most of our patients attended the day clinic once a month for one-day infusion therapy with iloprost; they also received paraffin baths and physiotherapy. SF-36 scores poorer than those of our SSc patients were found in a study in which the majority of patients were outpatients and members of patient associations [13]. This might have been due to the higher percentage of diffuse SSc (36%) in that study. Rannou et al. also reported a large percentage of diffuse SSc (46%) in 50 SSc patients in the French patient association who did not differ substantially in terms of their SF-36 items from the study by Mestre-Stanislas et al. with more than 50% diffuse SSc, but also had a poorer QoL scores than ours in all parameters [10]. In comparison to our patient group, 80% vs. 15% of those patients had arthralgia and 86% vs. 37% gastrointestinal involvement.

Hudson et al. compiled SF-36 scores for 504 SSc patients [3]. The SF-36 subscale and summary scores for SSc were significantly poorer than those for the general US population, except for mental health and the mental component summary score. In all, their scores were worse than those of our patients. The patients were similar to ours as regards the percentage of women (86% vs. 88%) and mean age (56 vs. 58 years). However, 44% patients had diffuse SSc, compared to 5% of patients in our group.

In a cross-sectional multicenter study consisting of 416 patients from the Canadian Scleroderma Research Group Registry, the authors evaluated the patients’ self-reported physical health [8]. Thirty-five percent had diffuse SSc. Their mean Rodnan score was 10.6, compared to 6.7 in our group. Additionally, shortness of breath, gastrointestinal problems, and swollen joints were significant clinical predictors of impaired QoL. In our study, 41% of patients had lung involvement and 85% had skin involvement. Dysphagia and gastrointestinal disease were seen in 37% of our patients.

Differences in control populations and variations in QoL from country to country could also explain why all the other QoL studies on SSc produced poorer SF-36 scores than ours. Our control group, the healthy German population (mean age 47.7 years), showed better subscale scores in all dimensions but in physical role compared to a healthy control group in Naples [12]. In the US control population norms (women aged 45-54 years), all SF-36 scales were substantially poorer than in our controls [3].

SSc patients have significantly higher subscale scores in mental health and social function than those with other chronic inflammatory dermatoses. Since the majority of patients with SSc have progressive disease, they are unlikely to experience an improvement in QoL. SSc patients might be able to cope better with the disease and so have better scores in the mental components. Besides, SSc as a chronic condition might be better tolerated than a disease like atopic dermatitis, which can feature stress-induced flares.

In SLE, QoL was even poorer than in patients with hypertension, diabetes or myocardial infarction [26]. SSc and SLE have a similar course of disease and are more common in younger or middle-aged women. In patients with non-melanoma skin cancer, QoL correlated significantly with the patients’ coexisting illnesses and medical risk factors [17]. Stigmatization because of rash in SLE, and sclerosis of the face and hands in SSc, the uncertain course of the disease, fatigue, environmental factors (such as sun exposure in SLE and cold in SSc), and reduced life expectancy are some of the factors that influence QoL. Besides the parameters evaluated on the SF-36 questionnaire in SSc, disease burden [30], body appearance, stigmatization [31] and impaired hand function are of similar magnitude as in patients with rheumatoid arthritis [32]. As indicated by Nguyen, the aesthetic appearance is important in all dermatologic diseases with visible skin lesions such as in SSc [29]; but chronic eczema on the face, psoriasis, ichthyosis, pemphigus, nail disease, or vitiligo can also invoke negative emotions and result in reduced self-esteem [21, 33]. Moreover, chronic diseases such as SSc may cause frequent absences from work, as is also the case in those diseases that feature itching, skin rash flares, pains or fatigue (contact eczema, atopic dermatitis, arthralgia/arthritis in psoriasis, lupus erythematosus).

Limitations of the study were the low prevalence of diffuse SSc compared to other studies, which impeded an exact comparison of data. This was not caused by selection bias since we investigated consecutive SSc patients. The fact is that we mostly care for patients who have limited SSc or uSSc since patients with diffuse SSc might more often be seen at the rheumatology department. But we have clearly shown that dyspnoea or different hand disabilities impair QoL. Another limitation was the small number of patients and that fact that we only used the SF-36 questionnaire to determine QoL, although there are other instruments that measure disability such as the scleroderma Health Assessment Questionnaire HAQ [10], the Cochin Hand Function Scale (CHFS) [34] and the Scleroderma Assessment Questionnaire (SAQ) [11]. We could not include all the studies using the SF-36 in diverse dermatologic diseases that are available from PubMed but we showed examples to underscore disease burden in the different dermatologic diseases.

## Conclusion

SF- 36, as a patient self-assessment instrument, cannot distinguish between the different SSc subtypes but does reflect organ involvement that can cause disabilities affecting the SF-36 subscales. Clinical factors such as digital ulcers, dysphagia, or dyspnoea can be measured by laboratory or clinical methods. Non measurable factors are burden of disease, degree of stigmatization, or hand disability, which represent the sum of physical and psychological factors that all contribute to the empirical dimension we call quality of life.

Our results indicate that SSc has high disease burden as compared to the general population and to patients with more common skin diseases. Since SSc is a rare disease, there is a lack of awareness on the part of health care providers and policy makers. This is unfortunate for the sufferers since their QoL is much lower than that of patients with more common chronic conditions such as heart disease or diabetes [3].

Unlike psoriasis, atopic dermatitis, pemphigus, or SLE, remission cannot be achieved with SSc. Patients can adapt well to their slowly progressing disease so that social factors and disease coping may contribute to relatively better mental health than in other dermatoses. More therapeutic emphasis should therefore be placed on techniques for coping with the disease and biopsycho-social support [35, 36]. When it is an option for the patient, spiritual care can improve mental health scores by helping to alleviate depression or pain [37, 38]. Clinical practice should feature an interdisciplinary approach to SSc patients, involving physicians, physical and occupational therapists, psychologists, social workers and spiritual advisers. The influence of biopsycho-social-spiritual supportive interventions could be an area for fruitful future study.

## Methods

### Patient selection

In 2012, 45 consecutive patients with SSc who had regular outpatient or day clinic appointments were enrolled at the Department of Dermatology for complete medical workup; the detailed clinical data were to be the basis of a diploma thesis (Painsi C. Dermatologic markers of systemic sclerosis in pulmonary hypertension, 2009-2010). This study was approved by the Ethics Committee of the Medical University of Graz.

Forty-one patients agreed to fill out the SF-36 questionnaire. This was part of data acquisition program for the German Network of Systemic Sclerosis (DNSS). Based on ACR criteria, the patients were classified into 29 with limited cutaneous SSc, 2 with diffuse SSc, and 10 with uSSc [2]. USSc was diagnosed in patients who did not fulfill the ACR criteria but suffered from early SSc, defined by a positive Raynaud’s phenomenon (RP) and at least one further feature of SSc: positive nail fold capillary alterations, puffy fingers, or detectable scleroderma-associated autoantibodies [2, 39].

The patient’s history was taken, including duration of RP and skin sclerosis. Pulmonary function was determined with diffusing capacity of the lung for carbon monoxide (DLCO); stress echocardiography and right heart catheterization were performed to diagnose or rule out pulmonary hypertension (PAH). Oesophagus dysmotility was shown by barium swallow, kidney function by serum creatinine levels and urinalysis. Patients were examined for the extent of skin sclerosis, digital ulcers, scars and calcinosis, and queried for arthralgia and arthritis. The Rodnan score and Karnofski index were documented.

In 2012 we searched PubMed for studies on QoL in SSc and in chronic skin diseases measured by the SF-36 questionnaire and used their data, when appropriate, for comparison.

### QoL assessment and function measures

The SF-36 questionnaire comprises eight scales: physical function, physical role, bodily pain, general health, vitality, social function, emotional role, and mental health. For each scale, the score ranges from 0 (poor) to 100 (excellent) to define health status. Scores are also summarized into two global scores, i.e. the physical component score (PCS) and the mental component score (MCS) [14].

For global assessment, these 8 scales were evaluated in all 41 SSc patients and were compared with published data obtained from the normal population [14], patients with SSc [3, 7, 8, 10, 13], patients with occupational contact dermatitis [15], ichthyosis [16], non-melanoma skin cancer [17], eczema depending on the body site [18], atopic dermatitis [19, 23], nail disease [20], vitiligo [21], work-related skin diseases in health care workers [22], psoriasis [24], pemphigus [25], systemic lupus erythematosus (SLE) [26], and other chronic skin disease [14].

### Statistical analyses

Data are presented as mean and standard deviations. For statistical analysis a two-sided one-sample t-test was used to determine significant differences between our patients’ mean values and published mean values from other SSc patient groups, other skin diseases, and the normal population without chronic diseases. When a normal distribution could not be assumed, nonparametric tests were performed. To analyse clinical factors that could influence QoL, we used the chi square or Fisher’s exact test for categorical data and Spearman rank correlation for continuous data. No multiple testing p-value adjustment was made since this was an exploratory analysis [40]. A p-value less than 0.05 was considered statistically significant. Data were analyzed using the statistical software IBM SPSS Statistics (Release 20.0.0. 2011. Chicago (IL), USA: SPSS Inc., an IBM Company).

### Ethics

Data acquisition was approved by the Ethics Committee of the Medical University of Graz.
